# Serum levels of the angiogenic factor pleiotrophin in relation to disease stage in lung cancer patients

**DOI:** 10.1038/sj.bjc.6600202

**Published:** 2002-03-18

**Authors:** R Jäger, B List, C Knabbe, B Souttou, D Raulais, T Zeiler, A Wellstein, A Aigner, A Neubauer, G Zugmaier

**Affiliations:** Philipps University of Marburg, Center of Internal Medicine, Department Hematology/Oncology Baldingerstr., 35033 Marburg, Germany; Philipps University of Marburg, Department Transfusion Medicine and Haemostaseology, Baldingerstr., Marburg, Germany; Robert-Bosch-Krankenhaus, Department Clinical Pathology, Stuttgart, Germany; Georgetown University, Lombardi Cancer Center, Reservoir Road, Washington, D.C., USA; Institute d'Oncologie Cellulaire et Moleculaire Humaine, Bobigny, France; Differentiation and Cell Proliferation, INSERM, U440, 75005 Paris, France; Philipps University of Marburg, Department Pharmacology, Max v. Frisch Str., Marburg Germany

**Keywords:** NSCLC, SCLC, pleiotrophin, VEGF

## Abstract

Pleiotrophin is a heparin-binding growth factor involved in the differentiation and proliferation of neuronal tissue during embryogenesis, and also secreted by melanoma and breast carcinoma cells. Pleiotrophin exhibits mitogenic and angiogenic properties and has been shown to influence the vascular supply, expansion and metastasis of tumour cells. Our aim was to study the serum and plasma concentrations of pleiotrophin and the classical angiogenic growth factor vascular endothelial growth factor. Using a specific ELISA-test we studied patients with small cell lung cancer (*n*=63), and patients with non-small cell lung cancer (*n*=22) in comparison to healthy control subjects (*n*=41). In most of the lung cancer patients (81%), we found serum levels of pleiotrophin above those of control subjects (*P*<0.001). Of the 63 small cell lung cancer patients in the study pleiotrophin serum levels were elevated in 55 cases (87%) and in 14 cases (63%) of the 22 non-small cell lung cancer patients. Pleiotrophin mean serum concentrations were 10.8-fold higher in the tumour patient group as compared to the control group (*P*<0.001). Furthermore, pleiotrophin serum levels correlated positively with the stage of disease and inversely with the response to therapy. Plasma vascular endothelial growth factor concentrations were elevated in only in 28.6% of small cell lung cancer and 45.5% of non-small cell lung cancer patients by an average of 2.3-fold. Quite strikingly, there was no apparent correlation between the plasma vascular endothelial growth factor concentration and the stage of disease. Our study suggests that pleiotrophin may be an early indicator of lung cancer and might be of use in monitoring the efficacy of therapy, which needs to be confirmed by larger studies.

*British Journal of Cancer* (2002) **86**, 858–863. DOI: 10.1038/sj/bjc/6600202 www.bjcancer.com

© 2002 Cancer Research UK

## 

The development of a neovascular network, a process known as angiogenesis, has been shown to be important in the growth of a variety of human solid tumours, including lung cancer. Angiogenic factors, produced either by tumour cells or by non-tumour cells within the tumour microenvironment, act in concert with angiogenesis inhibitors to regulate the process of tumour neovascularisation. A number of factors which promote the migration of vascular endothelial cells have been identified and include pleiotrophin (PTN) and vascular endothelial growth factor (VEGF) (Connolly *et al*, 1989; Fang *et al*, 1992; Zhang and Deuel, 1999; Kuroi and Toi, 2001; Souttou *et al*, 2001).

Vascular endothelial growth factor (VEGF) is a very effective factor in inducing the formation of new blood vessels, and acts specifically on endothelial cells. VEGF is expected to play important roles in inflammation and during normal and pathological angiogenesis, a process that is associated with wound healing, embryonic development, and growth and metastasis of solid tumours (Brown *et al*, 1992; Collins *et al*, 1993). VEGF is a homodimeric 34–42 kDa, heparin-binding glycoprotein and is expressed by almost all human solid tumours (Senger *et al*, 1986).

Pleiotrophin (PTN) (Li *et al*, 1990), also referred to as heparin-binding growth-associated molecule (HB-GAM) (Merenmies and Rauvala, 1990), is a growth factor which is involved in the differentiation and proliferation of neuronal cells during embryogenesis (Merenmies and Rauvala, 1990; Bohlen and Kovesdi, 1991). Pleiotrophin is a very basic protein of an apparent mass of 18 kDa, which is differentially expressed during pre- and postnatal development (Hampton *et al*, 1992). During embryogenesis PTN is strongly expressed in brain, liver, spleen, lung, bone and tongue. Only weak expression of PTN is found in adult brain, liver, bone and tongue, with low to no expression in other organs (Merenmies and Rauvala, 1990; Vanderwinden *et al*, 1992).

Elevated PTN expression has been shown to occur in malignant tumours with respect to normal tissue, for example in ovarian carcinoma, and in tumour cell lines such as breast carcinoma and glioblastoma (Wellstein *et al*, 1992; Nakanishi *et al*, 1997). A recent study investigated PTN in the serum of patients with various cancers and the results indicated that PTN is associated with the presence of tumour (Souttou *et al*, 1998; Kuroi and Toi, 2001).

We have previously shown that PTN mRNA is strongly expressed in human lung cancer cell lines, particularly in those cell lines derived from small cell lung cancer (Jäger *et al*, 1997). In the present study we have evaluated the serum concentration of PTN in patients with lung cancer in comparison with a control group of healthy subjects, using a modified highly sensitive enzyme-linked immunosorbent assay (Souttou *et al*, 1998). Furthermore, we compared the serum concentration of PTN in these patients with the plasma concentration of the classical angiogenic growth factor, VEGF, with respect to disease staging and response to therapy.

## PATIENTS AND METHODS

### Patients and healthy control subjects

Serum and plasma samples were obtained prospectively from 63 patients with histologically confirmed small cell lung carcinoma (SCLC) (University of Marburg, 52 males; median age 58 years). Twenty-three of the SCLC patients were staged as very limited disease (VLD) or limited disease (LD) (the tumour confined to one hemithorax and regional lymph nodes) and 40 patients were staged as extensive disease (ED) (tumour occurrence beyond these regions). Patients with extensive disease were subclassified into extensive I (no metastasis outside the lung), and extensive II (metastasis outside the lung) (EDI and EDII). Serum and Plasma samples were also obtained from 22 patients with non-small cell lung cancer (NSCLC) (median age 62 years; University of Marburg, Germany), 12 of whom were staged as IIb/IIIA and 10 of whom were staged as IIIB/IV. Forty-one healthy blood donors (University of Marburg) provided serum samples for the control group (25 male, median age 41 years).

Tumour specimens were obtained through biopsy by bronchoscopy in five lung cancer patients. The specimens were frozen in liquid nitrogen and stored at −80°C until analysis.

### RNA preparation and RT–PCR

RNA was isolated from tumour specimens by the RNAzol method (Cinna/Biotech, Houston, TX, USA). Briefly, samples were treated with 2 ml RNAzol and 200 μl chloroform, then centrifuged for 15 min at 15 000 r.p.m. The supernatant was carefully removed and diluted with the same volume of isopropanol. After centrifugation the pellet was washed twice with 75% ethanol and resuspended in 50–100 μl of distilled water.

### Reverse transcription

Total RNA (1 μg/7.5 μl) was denaturated at 70°C for 10 min. Denaturated RNA was reverse-transcribed in a 30 μl final volume of 5×RT buffer (10 mM Tris (pH 8.3), 50 mM KCl, 1.5 mM MgCl_2_), 625 μ of each dNTP, 2.5 RNasin (Boehringer Mannheim, Germany), 10 mM dithiothreitol and 5 U reverse transcriptase. The reaction mixture was incubated for 1 h at 37°C and stopped by an incubation for 10 min at 90°C.

### PCR

The cDNA (1.5 μl) was amplified in a 30 μl final volume of 10 mM Tris (pH 8.3), 50 mM KCl, 1.5 mM MgCl_2_, 200 μl of each dNTP, 0.5 μM of each primer and 1.25 U Taq polymerase (Boehringer Mannheim, Germany) for 25–30 cycles under the following conditions: 5 min at 95°C, 1 min at 56.5°C (PTN) or at 58°C (GAPDH) and 2 min at 73°C. GAPDH was used as a control. PCR products were loaded on a 1.5% ethidium bromide-stained agarose gel. Primer sequences were as follows: PTN, sense 5′ GGT CTC GAG TAT GTT CCA CAG GTG ACA TC 3′ and anti-sense 5′ GGT AAG CTT AGA GGA CGT TTC CAA CTC AA 3′; GAPDH sense 5′CGT CTT CAC CAT GGA GA 3′ and anti-sense 5′ GCC GGT AGT GCG GTG TCA AA 3′.

### Collection of blood samples, ELISA for PTN and VEGF

Blood samples were collected into EDTA-coated tubes and stored at 4°C overnight. Subsequently, samples were centrifuged to isolate serum and plasma. Haemolytic or lipemic samples were excluded. Aliquots of serum and plasma from each subject were stored at −80°C. ELISA results were not influenced by the duration of storage at −80°C, which ranged from 1 month to 4 years. The serum and plasma levels of PTN were determined by using a highly sensitive enzyme-linked immunosorbent assay as previously described (Souttou *et al*, 1998). In brief, a mouse anti-PTN monoclonal antibody (4B7) (Souttou *et al*, 1998) was diluted to 1 μg ml^−1^ in Tris-buffered saline (TBS). Aliquots (100 μl) of the diluted antibody were incubated in 96-well plates (Life Technologies, Germany) at 4°C overnight. Wells were washed three times with TBST (50 mM Tris-HCl (pH 7.5), 0.15 M NaCl and 0.5% Tween 20). Residual free binding sites were blocked with 200 μl TBST containing 1% bovine serum albumin for 2 h at 4°C, before washing the wells three times with TBST. Serum was diluted 1:1 with double concentrated TBST. One hundred microlitres of this dilution was added per well and incubated at room temperature for 1 h. The wells were then washed three times with TBST and the secondary antibody, a biotinylated affinity-purified anti-human PTN goat immunoglobin G (IgG) (R&D, Germany), was added at a concentration of 500 ng ml^−1^ and incubated at room temperature for 1 h. After washing three times with TBST, 100 μl of streptavidin-conjugated alkaline phosphatase (50 ng ml^−1^) was added per well and incubated for 1 h at room temperature. The plate was then washed three times with TBST and incubated with 100 μl of a p-nitrophenyl phosphate substrate in the dark at room temperature for 2 h. Absorbance was measured with a microtiter plate reader (Labsystems Multiscan, Frankfurt, Germany) at 405 nm. Recombinant human pleiotrophin (PTN) (R&D, Germany) served as control to provide a standard curve. Results were analysed using Genlite (Labsystems Multiscan). SAS (Cary, NC, USA) software was used for the statistical analysis.

In the second ELISA we used a human VEGF-immunoassay Kit from Quantikine (R&D Systems, Germany). This assay employs also the quantitative sandwich enzyme immunoassay technique. Serum and plasma levels of VEGF were determined.

## RESULTS

All samples were analysed for PTN and VEGF in serum and plasma. Serum and plasma levels of PTN were comparable (not shown). Therefore serum levels of PTN were used to show the data, in order to keep up consistency with results published previously (Souttou *et al*, 1998). Serum levels of VEGF turned out to be about five times higher than plasma levels. In order to avoid interference with VEGF released from platelets, the data were demonstrated by showing VEGF plasma levels. Therefore PTN serum levels were compared with VEGF plasma levels.

### Detection of pleiotrophin in serum of patients with lung cancer in comparison to healthy control subjects

In our study a group of 41 healthy blood donors 16 had pleiotrophin serum concentrations below the sensitivity of the ELISA assay. The maximum serum concentration measured in the control group was 3230 pg ml^−1^ and the average concentration was 830 pg ml^−1^. This is somewhat higher than a previous study (Souttou *et al*, 1998), most likely due to modifications of the ELISA assay and the collection and preparation of blood samples.

In Figure 1

**Figure 1:**
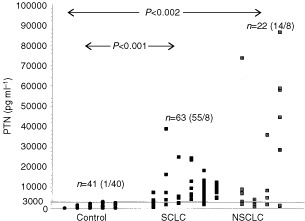
Serum concentrations of PTN. The numbers in brackets show the portion above and below the cut-off set as the highest value in the control group.

the serum concentrations of PTN in control subjects and lung cancer patients are shown. Our results show elevated serum levels in patients with SCLC and NSCLC with respect to the controls (*P*<0.001). In 97.6% of the healthy volunteers the serum concentrations of PTN were below 3000 pg ml^−1^. We used this as an arbitrary cut-off value for elevated PTN serum levels (>3000 pg ml^−1^). Under these conditions, serum concentrations of PTN were elevated in 81% of all investigated lung cancer patients, in 63% of NSCLC patients (*P*<0.002) and in 87% of SCLC patients (*P*<0.001). The mean PTN serum concentration were 10.8-fold greater in the tumour patients relative to controls.

### Disease stage relative to serum concentrations of PTN

**Figure 2:**
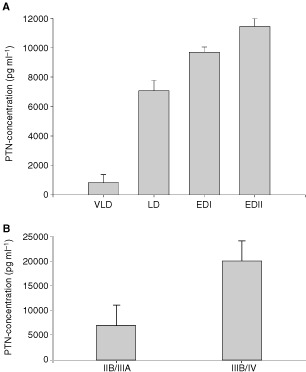
PTN serum concentrations according to stage of disease in patients with SCLC (**A**) and NSCLC (**B**).

The mean serum PTN concentrations in SCLC patients with extensive disease II (EDII) was 11431 pg ml^−1^ (*n*=26), EDI 9690 pg ml^−1^ (*n*=16), limited disease (LD) 7068 pg ml^−1^ (*n*=20) and in one patient with very limited disease (VLD) 814 pg ml^−1^ (*n*=1) (Figure 2A

). Statistical analysis showed a significant positive correlation between serum PTN concentration and stage of disease in SCLC patients.

Similarly, the serum concentration of PTN was found to increase with the disease stage of NSCLC patients. The mean serum PTN concentration was 6909 pg ml^−1^ (*n*=10) in patients with tumour stage IIB/IIIA and 19944 pg ml^−1^ (*n*=12) in patients with tumour stage IIIB/IV (Figure 2B). Patients with PTN serum levels above the median showed a median overall survival of 10 months. Survival of patients with PTN serum levels below the median ranged from 14 months to >2 years up to now (data not shown). Some of these patients are still alive at the present date. Due to the small number of patients statistical analysis did not reveal a significant difference.

### PTN serum concentration in patients relative to therapy

**Figure 3:**
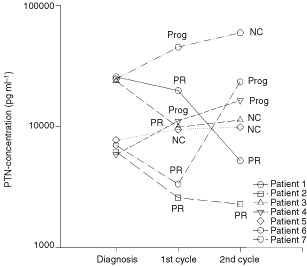
PTN serum concentration relative to the response to chemotherapy (*n*=7 patients), Serum samples were taken at the time of diagnosis and after each of two cycles of chemotherapy. PR=partial remission; Prog.=tumour progression; NC=no change

The serum concentration of PTN was monitored in seven patients from the time of diagnosis over the course of two cycles of chemotherapy with cisplatin in the first cycle and ifosphamide in the second cycle (Figure 3

). Two patients entered remission after one cycle of treatment. In these patients the PTN serum concentration decreased after both chemotherapy cycles. Three patients showed little clinical response to the first chemotherapy course, with progression of disease by the end of the second cycle. In these cases the serum PTN concentration either remained stable or rose further. Two patients showed partial remission but relapsed after the second cycle. Both patients showed an initial decrease in serum PTN concentration but a subsequent increase coincident with the relapse.

### Detection of VEGF in plasma of patients with lung cancer

**Figure 4:**
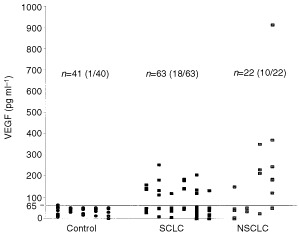
VEGF serum concentrations in control subjects, SCLC- and NSCLC patients.

In parallel with the measurements of PTN, we investigated the plasma concentration of VEGF in the same group of patients with lung cancer compared to control subjects (Figure 4

). The mean plasma VEGF concentration in the group of control subjects was 40 pg ml^−1^ compared to 91 pg ml^−1^ in the group of lung cancer patients (154 pg ml^−1^ in the NSCLC group and 69 pg ml^−1^ in the SCLC group), constituting a 2.3-fold higher concentration of VEGF in lung cancer patients overall. 45.5% of NSCLC patients and 28.6% of SCLC patients were above the cut-off value of 65 pg ml^−1^ established from the study in control subjects. No correlation between VEGF plasma concentration and histological diagnosis or stage of disease was found (data not shown).

### Comparison of PTN and VEGF expression in patients with SCLC and NSCLC

Since VEGF is a classical marker of angiogenesis, known to be elevated within the plasma of the majority of cancer patients, we have used it in our study for comparison with PTN. The serum PTN concentrations in each of the patients studied bore no correlation with the VEGF plasma concentrations (not shown).

### Expression of PTN mRNA in lung cancer specimens

**Figure 5:**
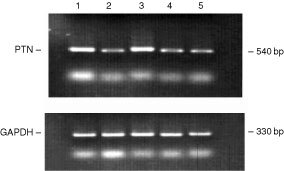
Pleitrophin (PTN) mRNA expression in tumour specimens of three small-cell-lung cancer (SCLC, lanes 1, 3 and 5) patients and two non-small cell lung cancer (NSCLC, lanes 2 and 4) patients as determined by RT–PCR (for details see Patients and Methods).

The expression of PTN transcripts was determined in tumour specimens obtained from five lung cancer patients. RT–PCR analysis revealed that all tumour specimens investigated expressed PTN-mRNA (Figure 5

).

## DISCUSSION

Since PTN is expressed by endocrine cells (Riegel and Wellstein, 1994), we were interested to investigate whether its expression is a feature of the neuroendocrine tumour SCLC and whether it can be detected within the serum of patients with this tumour. We have previously shown that in lung cancer cell lines PTN mRNA is predominantly expressed in 78% of SCLC cell lines compared to only 25% of NSCLC cell lines (Jäger *et al*, 1997).

The aim of the present study was to investigate in a prospective trial the potential merit of measuring concentrations of PTN in patients with lung cancer, by evaluating the distribution of this recently described angiogenic factor in comparison to that of the classical angiogenic growth factor, VEGF.

Our results reveal a distribution of elevated PTN serum levels in lung cancer patients that closely resembles the distribution of PTN expression in lung cancer cell lines (Jäger *et al*, 1997). Although the mean serum PTN concentration measured was greater in NSCLC than SCLC patients, it was elevated with respect to control subjects in a smaller portion of patients (63% of NSCLC *vs* 87% of SCLC patients). Since the age and sex ratios of the control subject group differed from the patient group, we age- and sex-matched 10 subjects from each of these two groups and found that neither age nor sex bore any influence on the serum PTN concentration (data not shown). These results suggest that PTN, as measured in serum by ELISA, may be a valuable marker of lung cancer, and in particular SCLC. Furthermore, the functional significance of PTN as a potent angiogenic factor in lung cancer clearly merits further investigation.

To our knowledge only one other study has reported elevated serum PTN concentrations in patients to date (Souttou *et al*, 1998). Souttou *et al* (1998) found significantly elevated PTN levels in patients with pancreatic carcinoma (*n*=41; *P*<0.0001) and colon cancer (*n*=65; *P*<0.0079) in comparison to 28 control subjects. Clearly, PTN has potential to serve as a marker of the presence of malignant disease, although it is unlikely to differentiate between tumour types. PTN overexpression in breast cancer cells induces increased vascular density *in vivo*, implicating an angiogenic role (Choudhuri *et al*, 1997). In primary human breast cancer, pleiotrophin expression is correlated with acidic FGF expression as demonstrated by Rnase protection analysis. Midkine a heparin-binding growth factor, which has a 45% sequence homology to PTN, shows a significant elevation of serum level in patients with various carcinomas (Relf *et al*, 1997; Ikematsu *et al*, 2000).

In addition to the clinically relevant finding that PTN is detectable in the serum of tumour patients, it is important to ascertain whether the source of PTN production is the tumour itself. Our result, that the serum PTN concentration in lung cancer patients correlates positively with the stage of disease, supports the hypothesis that PTN is produced by the tumour tissue. This hypothesis is further supported by our finding of PTN-mRNA expression in lung cancer specimens.

Finally, the serial measurements of serum PTN in seven patients undergoing chemotherapy showed significant reductions in PTN concentrations in those patients who responded clinically to treatment, but in none of those patients in whom disease progressed. In these latter patients PTN serum concentrations rose or remained constant. Similarly, Souttou *et al* (1998) found evidence of direct PTN secretion by tumour tissue. After the growth of implanted human PTN-expressing tumour cells within PTN-negative mice, PTN became detectable in the serum of the mice. Furthermore, after operative removal of the tumours the serum PTN concentrations in the mice returned to undetectable levels. The same results have been found in patients, when successful removal of pancreatic tumours led to a reduction in PTN serum levels (Souttou *et al*, 1998). Our data might give a preliminary indication for a longer survival of lung cancer patients with lower PTN serum levels as compared to the survival of lung cancer patients with higher PTN serum levels (not shown). However, larger studies are necessary to address this issue for a definite conclusion.

In the final section of our study, the plasma concentrations of VEGF were analysed for the same lung cancer patient and control groups to obtain a direct comparison with the data for PTN. A number of studies investigating VEGF serum levels in lung cancer patients have shown that the expression of VEGF does not correlate with either the histological type or the grade of lung cancer (Brattstrom *et al*, 1998; Takigawa *et al*, 1998) and our results are in agreement with these studies. Clearly, despite its potent angiogenic properties and acknowledged role as a marker of poor prognosis in various other malignant conditions (Nguyen, 1997), VEGF has only a limited role as a prognostic marker of lung cancer. In contrast, we show here that the measurement of serum PTN concentration may offer an effective marker of lung cancer, particularly of SCLC, with the possibility to monitor the response to therapy.

In conclusion our results indicate that PTN might be a prognostic factor for lung cancer and larger prospective further studies are required to confirm this hypothesis.
